# A cross-country analysis of climate shocks and smallholder food insecurity

**DOI:** 10.1371/journal.pone.0192928

**Published:** 2018-02-23

**Authors:** Meredith T. Niles, Jonathan D. Salerno

**Affiliations:** 1 Food Systems Program, Department of Nutrition and Food Sciences, University of Vermont, Burlington, VT, United States of America; 2 Environmental Studies Program, Sustainability, Energy, and Environment Complex, University of Colorado, Boulder, CO, United States of America; 3 Graduate Group in Ecology, University of California Davis, Davis, CA, United States of America; University of Maryland Baltimore County, UNITED STATES

## Abstract

Future climate changes will affect smallholder farmers in the developing world, posing threats to household food security. Nevertheless, there remains limited comparable evidence across multiple countries and regions regarding the global extent of climate shocks affecting smallholder food security. We examine data from 5,299 household surveys across 15 countries in Latin America, Africa and South Asia to assess the extent of climate shocks and their association with food insecurity, as well as what strategies may help buffer against climate shocks. We find that 71% of households reported experiencing a climate shock in the previous five years. Fifty-four percent reported experiencing food insecurity during one or more months annually. A multilevel statistical model estimated factors correlated with food insecurity as well as factors correlated with food insecurity among households that had experienced a climate shock. Households that reported experiencing a climate shock were 1.73 times more likely to be food insecure. As well, larger and poorer households were associated with higher odds of food insecurity while using pesticides, keeping large livestock, and being more educated are associated with lower odds of food insecurity. Among households that had experienced a climate shock, additional factors are correlated with lower odds of food insecurity when compared to otherwise similar households: use of fertilizers, pesticides, veterinary medicines, large livestock, and household assets. Together, these results demonstrate the extent of existing climate shocks affecting smallholder farmers and how interventions may potentially support adaptation and reduce food insecurity.

## Introduction

International development goals principally focus on eradicating poverty and food insecurity [1]. These goals shaped development objectives beginning in the Green Revolution through the Millennium Development Goals and remain central in the post-2015 Sustainable Development Goals (SDGs). The focus on food security is crucial for a number of reasons, including the wraparound benefits it has for achieving other development goals related to education, health and democracy [2,3]. Despite progress in recent decades, food insecurity continues to plague 815 million people globally, nearly two-thirds of whom are in Sub-Saharan Africa and South Asia [4], and half of whom are smallholder farmers and livestock keepers [5,6]. As we enter a new phase of SDGs, eradicating poverty and ensuring food security of a continuingly growing population remain central goals, made more challenging by uncertainty regarding future climate changes.

Climate change and climate shocks can cause food insecurity [7] and will become increasingly causal as extreme climate events grow more frequent and widespread [8,9]. The ways in which climate and extreme climate events affect agriculture are diverse and of varying scales [7]. It is expected that longer term warming trends will significantly influence crop yields, particularly in the tropics [10] over a multi-decadal time horizon. But climate “shocks” (i.e. events that outstrip the capacity of a society to cope with it, including events such as drought, floods, or heat waves [11][12]) present significant and immediate threats to household food security. For example, shocks can directly destroy crops or livestock or cut off roads and transport systems. Moreover, shocks can differentially affect households of the same area due to varying abilities to cope and adapt (e.g., farmers planting drought tolerant vs. non-improved seed, or having access to variable social safety nets from kin or institutions) [13,14], or shocks can universally affect communities or regions (e.g., a large-scale flood or drought event) [15]. Further complicating this issue, smallholder farmers in the developing world are likely to be in regions where climate impacts will be greatest, and food insecurity security already challenges livelihoods [16–18].

As climate shocks are estimated to increase under future climate scenarios and particularly affect those in low-income countries [19–21], it is important to assess how farmers have adjusted to previous shocks in order to predict future barriers to adaptation and potential supporting policies. Farmer adaptation has therefore become a focus of development goals and associated interventions [22], although recent research suggests that existing studies may tend to underestimate climate impacts for low-income countries [20]. Success in addressing the food insecurities present across smallholder households under climate shocks hinges on understanding both how household adaptation may occur and mechanisms for improved delivery of interventions to foster coping capacity and ultimately build resilience through adaptation [13,23]. Since the same climate event in one area may have profoundly different impacts on households of varying capabilities [24], the way households perceive a climate shock is an important lens through which to assess vulnerability and its potential impact on the household.

Operationalizing the SDGs and development action requires evidence-based knowledge for how to combat food insecurity, hunger, and malnutrition in light of emerging and future threats from climate change (among many others). Several bodies of work have presented frameworks, assessments and scenarios to review existing work or project potential food security challenges under different climate futures [25–27]. Projection models have also contributed scenarios of crop yield and production changes over multiple decades and inferred potential shifts in food security (e.g. [15,28–30]). However, most efforts have focused on potential agricultural yield impacts from climate change [31] while empirical evidence examining social and behavioral considerations has been less numerous [32,33] and mostly examined behavior changes related to adaptation practices and food security often through placed-based case studies [12,34,35].

Given the expected variability of climate impacts, one limitation of these micro-level efforts is that they cannot capture the ways in which climate change may cut across regions and determine how behaviors will vary under different climatic and socio-cultural contexts [36]. Though efforts have used multi-country analyses to examine how adoption of different practices influences food security [37] or factors (including climate) that correlate with smallholders’ adoption of adaptation behaviors [38,39], we are unaware of any large scale studies employing household-level empirical data to directly assess the relationships between climate shocks and food insecurity. This existing work lays the foundation to assess what the future may look like by understanding how climate shocks have already affected food insecurity, a question which currently lacks strong empirical evidence across many regions. Large-scale, comparative, evidence-based work is important to understand both why certain regions or areas see varying impacts and responses, but also to assess whether certain interventions can be scaled up across regions, which is particularly of interest to international development organizations and inter-governmental organizations.

Climate shocks or extreme weather events occur in different forms, cause variable impacts based on livelihood and biophysical context, and elicit variable responses from households based on perceptions of the shock and adaptive capacities [40]. The focus of this study is not on shock events themselves, or on perceptions of what constitutes a shock. Instead, we focus on the different features of household livelihoods, of which there are many [41], following a shock that enable adaptation in the form of reducing food insecurity. Following a climate shock, we assume that households able to access productive capacities to augment crop and livestock production and other forms of economic output are better suited to adapt to the subsequent higher likelihood of experiencing food insecurity. While this assumption is intuitive, we predict that ability to access productive capacities, as well as their ability to buffer households, will vary substantially by household context and by location. Understanding that agency is central component of household livelihoods, and of adaptive decision-making in response to climate change [13], directly informs our investigation.

Here we use a multi-country dataset across three continents and 15 countries to assess: 1) How are perceived climate shocks associated with food insecurity? and 2) What household characteristics and existing practices are correlated with reduced odds of food insecurity in the presence of climate shocks? We assess these questions by considering descriptive summaries of the global dataset and by fitting a multilevel statistical model predicting household food insecurity.

## Materials and methods

### Household data

**Fig 1:**
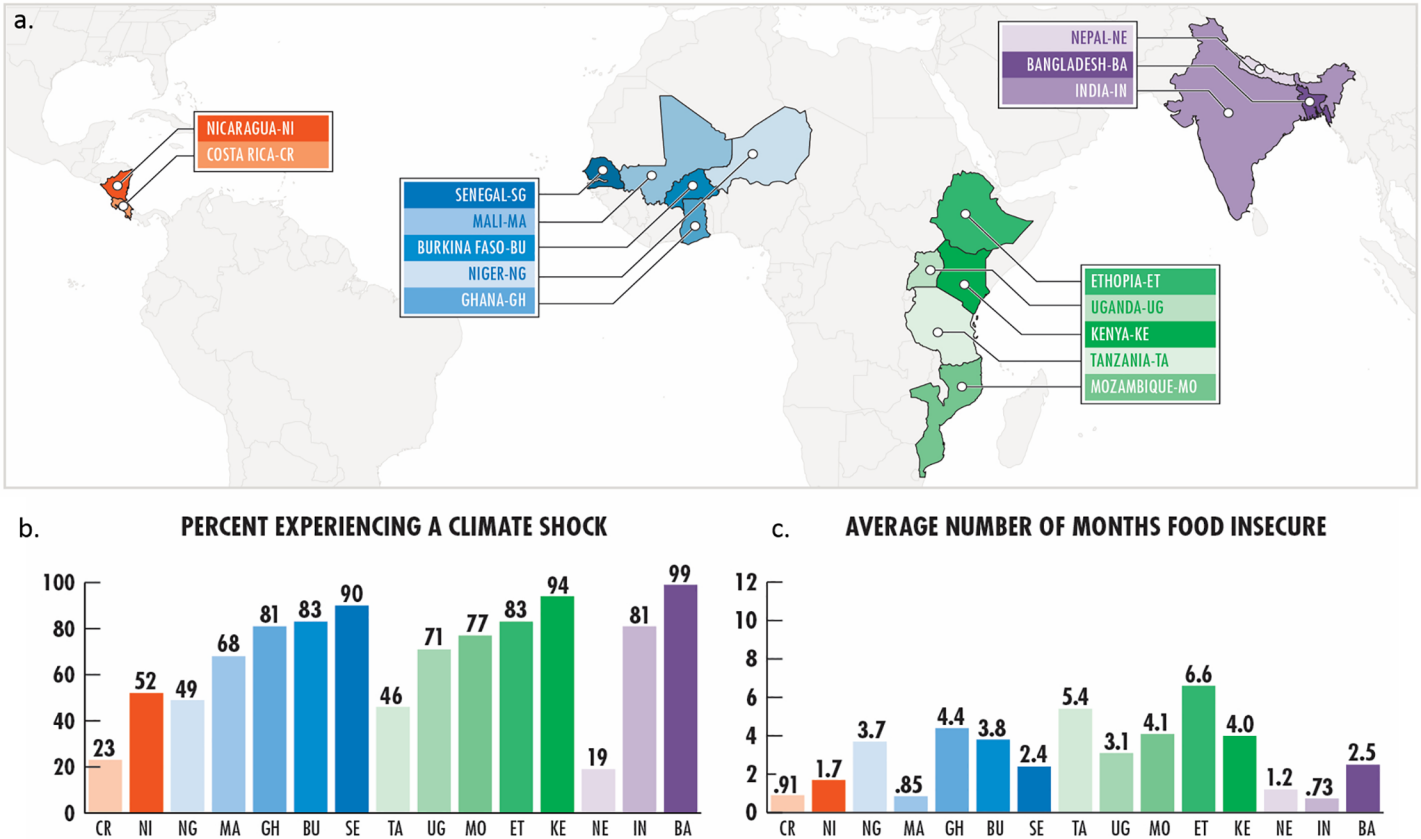
Country and region food insecurities and climate shocks. Households included in the study (Fig 1A) across four regions and 15 countries are color-coded by geographic region and shading represents intensity within the region scaled on the percent of households that faced a climate shocks (Fig 1B). Average rates of food insecurity are listed by country (Fig 1C).

CCAFS conducted household surveys during 2010–2012 to provide a baseline for monitoring and evaluating climate adaptation policies and interventions [42]). In each country, one to ten sites were defined by geographic boundaries, and approximately 140 households were randomly selected from within these site boundaries. Sites contained multiple villages, most commonly seven with 20 households selected per village, but the number of villages per site varied. Because the village-level sampling frame was not consistent across all sites, we adhere to the hierarchical sample structure of households clustered in sites and sites in regions (Figure A in S1 File). Regional designations are based in part on geography; also, Bangladesh, India, and Nepal exhibited notable differences in food insecurity outcomes (Figure A in S1 File) and in key household variables (Table 1), and so are separated in reporting descriptive data and in the model. In addition, Latin America contains only two countries, so while we report it as a region, it is important to note that our sample may be less representative than it is in other regions. Additional details on the specific methodological approach to the data collection can be found in Förch et al. [43]. Data describe 5,299 farming and livestock keeping households in 39 sites and 15 countries (Fig 1).

**Table 1:** Asset and demographic control variables in models. Regional designations are made based on aggregating sites and countries geographically, and also on differences in food insecurity and predictor variables (col. 1). Bangladesh, India, and Nepal are reported as separate regions. Note that some regions contain only one site.

Model Variable		Question	Averages (Percent)						
		*In the last 12 months did you…*	Global	Bangladesh	East Africa	India	Latin America	Nepal	West Africa
Productive Capacity Assets	Certified Seed	*use any purchased*, *certified/improved seed?*	46%	45%	45%	69%	20%	40%	29%
	Fertilizer	*use any purchased inorganic/mineral fertilizer?*	59%	63%	18%	78%	39%	100%	60%
	Herbicides/Pesticides	*did you purchase any pesticides/herbicides?*	56%	60%	35%	63%	78%	65%	42%
	Veterinary Medicines	*did you purchase any veterinary medicines?*	61%	67%	52%	58%	60%	66%	70%
	Credit	*get any credit for agricultural activities?*	15%	7%	7%	31%	8%	9%	16%
Animal Assets	Large Livestock	*produce from your own farm large livestock?*	58%	44%	42%	71%	44%	86%	62%
	Small Livestock	*produce from your own farm small livestock?*	69%	89%	81%	24%	79%	77%	91%
Demographic Controls	Single Female-Headed	*Household type*	10%	3%	30%	3%	20%	4%	4%
	Single Male-Headed		2%	3%	4%	1%	6%	1%	2%
	Primary Education	*What is the highest level of education obtained by any household member?*	38%	28%	51%	29%	48%	20%	53%
	Secondary Education		33%	40%	29%	34%	35%	42%	21%
	Post-Secondary Education		19%	29%	8%	26%	12%	35%	1%
	Household Size (average)	*How many people*, *including yourself and other adults*, *are in your household*	6	5	5	7	5	7	7
Shock	Climate Shock	*Have you faced a climate related crisis (eg*. *Flood*, *drought*, *frost*, *tidal surge) in the last 5 years?*	71%	99%	74%	81%	38%	19%	74%

We define focal variables from household data to use in assessing our research questions (Table 1). Food insecurity is measured as the total number of months in a typical year during which respondents reported not having adequate food for their household [37]. This is not a clinical measure of food security; it is the perceived level of food security within the home, a limitation we describe further in the discussion. Households reported whether they experienced a climate crisis (encompassed within our definition of “shock”) during the last five years. Here, we describe livelihoods through productive capacity and demographic control variables. We created an index of household wealth by applying the Gifi method of non-linear principal components analysis [44] to a set of 24 binary indicators of durable asset ownership (e.g., radio, mobile phone). In order to account for variation in asset value across the global study area (see [38], we calculated site-specific quintiles of the continuous wealth index [45] and then assigned a binary indicator of rich and poor to households in the 20% highest and lowest site-specific wealth quintile, respectively.

### Statistical model

We fitted a single multilevel varying effects (i.e., hierarchical, mixed, random effects) regression model to test the associations among food insecurity, climate shocks, and a suite of predictor variables. The logit model estimated food insecurity as a binomial response with 12 trials, using a household-level varying intercept effect allowing for a household-specific adjustment to the baseline log-odds of ‘successes’ for each monthly food insecurity trial. The log-odds of food insecurity response are estimated as

$${logit(p}_{hsr})=\alpha +\beta x_{hsr}+H_{h}+S_{s}+R_{r}$$

where *p* is the probability of food insecurity; *α* is the shared intercept; ***x*** is a vector of covariates for household *h* in site *s* in region *r*, and *β* is a vector of corresponding slope parameters; *H*, *S*, and *R* are the varying intercepts for household, site, and region, respectively. This approach suitably predicts over-dispersed outcomes (e.g., 11 and 12 months of food insecurity). India, Bangladesh, and Nepal are defined as separate regions (as stated above; also, Figure A in S1 File and Table 1). The higher order varying effects control for unobserved factors affecting differences in food insecurity shared within sites and regions [46]. We interpret coefficient estimates of household-level predictors in terms of their multiplicative effect on the odds of a household experiencing increased food insecurity, and report 95% credibility intervals throughout. Varying intercept effects at the region and site levels are reported in Supplementary Materials.

## Results and discussion

Evidence from household data demonstrates the widespread experience of household food insecurity and perceived climate shock (Fig 1). Overall, 71% of households experienced a climate shock in the last five years. The prevalence of shock was highest in Africa where 74% of households on average had experienced a climate shock in the past five years (Fig 1B), although individual countries in our dataset reported much higher prevalence (e.g. Bangladesh at 99%, Kenya at 94%, Senegal at 90%). Fifty-four percent of households reported one or more months of food insecurity in a typical year with a global mean of 2.3 months of food insecurity. Food insecurity rates also had large variation ranging from less than one month (0.73- India, 0.85-Mali, 0.91-Costa Rica) to greater than 6 months annually in Ethiopia (Fig 1C). Again, Africa at a regional level experienced the greatest average rates of food insecurity (3.5 months), and East Africa therein (4.3 months). This variability of both food insecurity and climate shocks experienced within most sites, suggests that the impacts of climate events may be largely dependent on household-scale factors (such as assets and demographics reported in Table 1) and households’ ability to cope.

The statistical model estimates that perceived climate shocks are positively associated with increased food insecurity, such that the experience of climate shock increases the odds of experiencing more severe food insecurity by a factor of 1.73 [1.24–2.36]. This effect is averaged over all households in the global sample and accounts for unobserved variation in food insecurity between sites and between regions (Figure A in S1 File).

A significant increase in the odds of more severe food insecurity is also associated with owning few durable assets (1.71 [1.33–2.16]) and larger households (1.02 [1.00–1.05]). Odds of reduced food insecurity are associated with pesticide use (0.65 [0.51–0.83]) and large livestock (0.50 [0.38–64]). Among household control variables only education also has a significant effect to reduce odds of food insecurity at the secondary (0.78 [0.64–0.94]) and post-secondary level (0.47 [0.36–0.58]).

**Fig 2:**
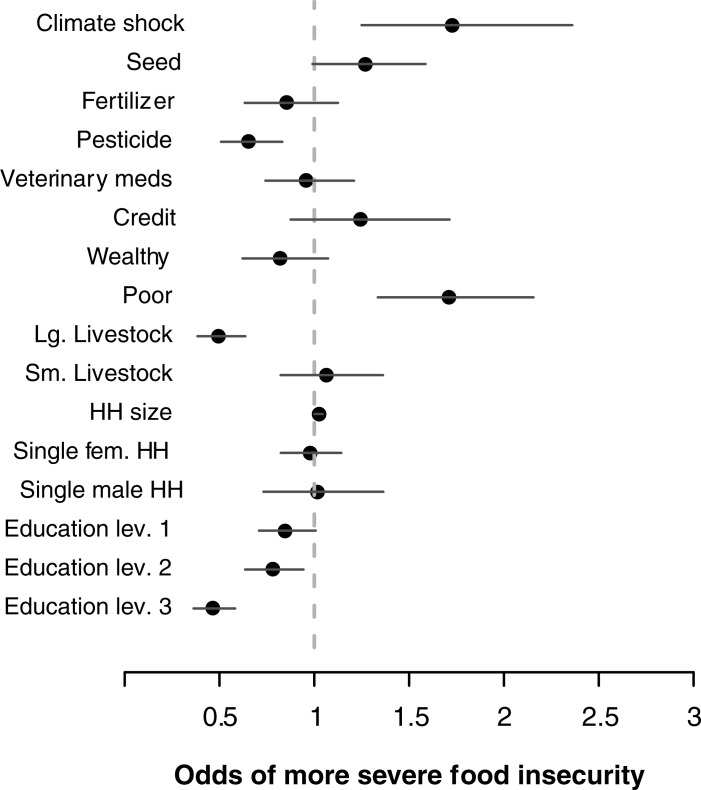
Main effects estimates from the multilevel model. Coefficient estimates are reported on the odds scale with 95% credibility intervals. The indifference value of 1 on the x-axis (dashed line; odds = 1/1) is equivalent to no effect. The model includes interaction effects and varying intercept effects for site and region (Figure A in S1 File).

**Fig 3:**
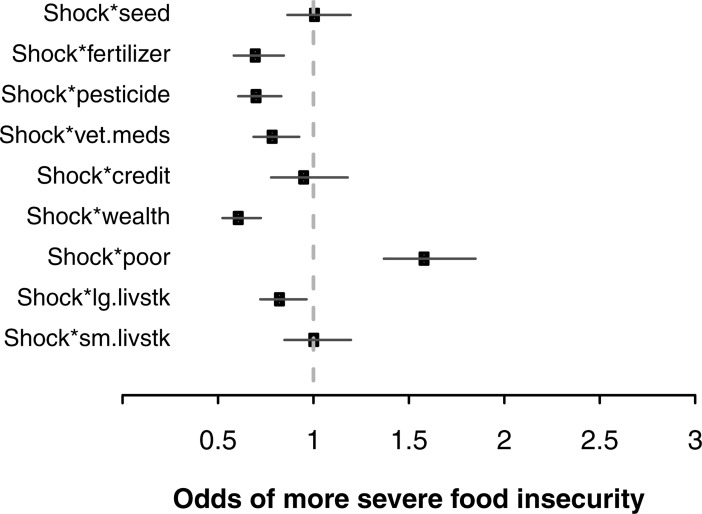
Effects of factors predicting household food insecurity among climate shocked households. Plotted estimates represent the marginal effect on food insecurity associated with particular household-level predictors (95% CI), as compared to a hypothetical household that does not exhibit that predictor yet also experiences shock and is otherwise similar in regards to all other model variables. Plotted estimates are the additive results of corresponding direct (Fig 2) and interaction effects (Table 1 in S1 File). The model includes varying intercept effects for site and region (Figure A in S1 File), which are not displayed.

In addition to the main effect estimates noted above (also Fig 2, Table 2), the statistical model includes interaction effects between covariates of interest and the experience of a climate shock (Fig 3, Table 3). Because models with many main and interaction effects are difficult to interpret, we present graphs that allow the comparison of hypothetical household scenarios [47,48]. Among otherwise similar households who both experience a shock, Fig 3 (and Table 3) presents the effect of a single covariate (e.g., among two shocked households, one household uses improved seed while the other does not) on the odds of more severe food insecurity. These comparisons demonstrate the following: among households experiencing shock, those also using fertilizer (0.71 [0.58, 0.84]), pesticides (0.71 [0.61, 0.83]), veterinary medicine (0.80 [0.69, 0.93]), and owning durable assets (0.62 [0.52, 0.72]) and large livestock (0.83 [0.72, 0.96]) were associated with decreased odds of food insecurity as compared to households also experiencing shock yet not exhibiting these characteristics. Households experiencing shock and owning fewer durable assets (1.59 [1.37, 1.85]) were associated with increased odds of food insecurity as compared to households also shocked yet not exhibiting these characteristics.

**Table 2:** Main effects from multi-level hierarchical random effects models. Positive log odds indicate greater odds of food insecurity, while negative log odds indicate greater odds of food security. Results that are statistically significant within a 95% credibility interval are bolded for emphasis.

Variable	TE	Log Odds	95% Confidence Interval	
			Lower Bound	Upper Bound
**Climate Shock**	**1.73**	**0.532**	**1.249**	**2.361**
Seed	1.27	0.231	0.990	1.586
Fertilizer	0.85	-0.169	0.633	1.125
**Pesticides**	**0.65**	**-0.432**	**0.508**	**0.831**
Veterinary Medicines	0.96	-0.054	0.742	1.210
Credit	1.24	0.202	0.875	1.713
Wealthy	0.82	-0.211	0.622	1.073
**Poor**	**1.71**	**0.530**	**1.335**	**2.156**
**Large Livestock**	**0.50**	**-0.708**	**0.384**	**0.636**
Small Livestock	1.06	0.052	0.822	1.362
**Household Size**	**1.02**	**0.023**	**1.001**	**1.045**
Single Female-headed	0.98	-0.027	0.823	1.142
Single Male-headed	1.02	0.002	0.732	1.364
Primary Education	0.85	-0.171	0.708	1.007
**Secondary Education**	**0.78**	**-0.253**	**0.635**	**0.943**
**Post-Secondary Education**	**0.47**	**-0.770**	**0.363**	**0.583**

**Table 3:** Summative interaction effects (e.g. considered in the context of main effects) from multi-level hierarchical random effects models. Positive log odds indicate greater odds of food insecurity, while negative log odds indicate greater odds of food security. Results that are statistically significant within a 95% credibility interval are bolded for emphasis.

Summative Interaction	TE	Log Odds	95% Confidence Interval	
			Lower Bound	Upper Bound
Climate Shock*Seed	1.02	0.01	0.86	1.19
Climate Shock*Fertilizer	**0.71**	**-0.35**	**0.58**	**0.84**
Climate Shock*Pesticides	**0.71**	**-0.34**	**0.61**	**0.83**
Climate Shock*Veterinary Medicines	**0.80**	**-0.23**	**0.69**	**0.93**
Climate Shock*Credit	0.96	-0.04	0.78	1.18
Climate Shock*Wealthy	**0.62**	**-0.48**	**0.52**	**0.72**
Climate Shock*Poor	**1.59**	**0.46**	**1.37**	**1.85**
Climate Shock*Large Livestock	**0.83**	**-0.18**	**0.72**	**0.96**
Climate Shock*Small Livestock	1.01	0.01	0.85	1.20

This paper set out to investigate the degree to which climate shocks are associated with food insecurity and what types of assets and interventions may best safeguard smallholder households against food insecurity in the presence of climate shocks. We demonstrate the association between climate shocks and food insecurity and the potential impacts they pose to smallholder farmers and livestock keepers globally. Seventy-one percent of households sampled perceived experiencing a significant climate shock during the previous five years, and we estimate these shocks were associated with an increase in the odds of food insecurity by a factor of 1.73. Among otherwise equal households that both experienced a shock, fertilizers, pesticides, veterinary medicines and large livestock are associated with decreased food insecurity. Unsurprisingly, those households with greater or fewer durable assets are associated with decreased or increased food insecurity, respectively, as many others have found (e.g. [49].

Our results suggest several important implications for development interventions in a changing climate. First, climate shocks matter- they are significantly associated with food insecurity. This indicates that as many have assumed, but not necessarily empirically demonstrated, if our future climate holds more climate shocks without adequate coping and adaptation abilities, it is likely that food security will be compromised among smallholders. Indeed, future projections for climate change include not only an increase in global temperatures, but a growing number of climate shocks, which have already affected global crop and livestock systems and food security. Future projections further assert an impact for food systems, particularly for food prices and in low-latitude areas [50] and could have significant implications for migration [51].

Second, irrespective of a climate shock, we provide evidence that pesticides, large livestock and education are associated with reduced odds of food insecurity. The use of pesticides or herbicides in agricultural development may support higher yields and reduced yield variance, the latter being particularly important in varying environments and with limited capacity for additional inputs. While there is certainly evidence of challenges with such inputs in agriculture (e.g. [52,53]), in an agricultural development context, opportunities to minimize weed and pest infestations can likely provide yield benefits, though it should be noted that non- chemical opportunities including push-pull technologies have also proven to be significantly positive for yield in smallholder contexts without negative environmental externalities [54]. Potential investment in these types of agricultural-based adaptations could also provide potential future mitigation benefits, if such investments reduce land clearing associated with agriculture by increasing yields [55]. Livestock for smallholder farmer food security has also been largely demonstrated in other literature, both for its potential to diversify diets as well as its use as an insurance mechanism [56,57]. Finally, the role of education here is critical to note in light of the SDGs, suggesting that there are co-benefits between education, agricultural development and food security, highlighting the interconnectedness of achieving the SDGs. The role of education for improving food security has been studied in other contexts, with potential diverse mechanisms. Wood et al. (2014) found that higher education levels was correlated with the use of new technologies or practices at a farm level[38], thereby leading to better production outcomes. However, education may also enable a household to seek outside employment and diverse income streams, which could also improve food security[41].

Third, we find that certain factors are correlated with decreased odds of food security in the presence of a climate shock, even though some of these factors are not associated with decreased odds of food security irrespective of a climate shock. For example, both pesticides and large livestock are associated with decreased odds of food insecurity both overall and in the presence of a climate shock. However, the use of fertilizers, veterinary medicines and assets are only significantly correlated with decreased odds of food insecurity in the presence of a climate shock. It may be that such counter-intuitive results (i.e. why would these only matter in a climate shock) could be the result of the way climate shocks are perceived, which we discuss further in our limitations section below, though we note that others have also had challenges with interpreting results in the same context [40]. In the context of a climate shock, the use of fertilizers in the system is not necessarily intuitive; however, we suggest that this is likely related to the ability of such inputs to increase yields overall, thus providing a greater base of agricultural production despite a climate shock. Further, with increased yields come additional crop residues, which can provide livestock feed, potential fuel, and soil conservation benefits [58]. It should be noted that such yield gains are not only possible with synthetic fertilizers; indeed, evidence suggests that organic and agroecological practices have had positive effects on yield and food security in Africa [59]. The role of veterinary medicines has not been widely discussed in the literature, but we suggest it may be critical for future considerations. Given the strong role that research suggests livestock can play in reducing food insecurity, such livestock may be susceptible to new diseases and future climate impacts. Ensuring that animals can be bred for resistance to future climate-induced changes is an emerging field of research [60] and will be critical for future adaptation among the nearly one billion livestock keepers globally [61]. Further, access to livestock vaccines and medicines, though not an explicit part of the data we examined, may be associated with access to veterinary medicines and could be important with the likely greater prevalence of pests and diseases [62]. Thus, our work demonstrates that merely having livestock, while important for food security, may not be enough to help safeguard smallholder farmers from future climate shock impacts on livestock.

Finally, we think it’s important to consider the variability present throughout this dataset. While we analyzed data at the global level, we controlled for the variability within regions and sites. In many cases, this variability was significant and demonstrates that perceived climate shocks within actual villages are not uniform. This is likely the result of the fact that data was collected on individual level perceptions of climate shock, which in fact may play a larger role in affecting households than has previously been considered. While development and assistance programs typically focus on major events like droughts and floods that may affect entire villages, it may be the case that smaller-scale climate shocks are influencing households more frequently, and for the particularly vulnerable, having significant effects on household food security. As such, assessments that quantify household-level perceptions of climate shocks should complement data tracking merely the incidence of these events.

Despite these results, this study also has notable limitations. First, given that these data are cross-sectional, our results suggest associations, and can be not inferred as causal. Future surveys across these same regions and households could provide a robust and important dataset that could enable greater causal analyses and we recommend that this be a priority for future work. Second, our results demonstrate some potentially counter-intuitive results, which may be the result of limitations in how survey questions were asked or interpreted. This may be particularly true for the food security measure, which was a perception of household food security based on a 12 month recall, rather than actual data on consumption. As a result, it may be possible that survey respondents discounted the impact of agricultural practices in the absence of a shock, which may explain why, for example, fertilizer use had no significant relationship to food security in the absence of a shock. Future research could aim to explore more data-driven approaches to monitoring and accessing food security, which while challenging in low-income country contexts, could yield greater insight. However, we also believe that the way in which climate shocks were asked as binary questions, without further detail, also may mask variation in how households experience different kinds of shocks and how the relative magnitude of a shock could have significantly different impacts. Future work in this area could combine this data with historical climate data to explore these relationships further, and future surveys could integrate more detailed questions about shock type and magnitude to explore these relationships further.

## Conclusion

As we enter a new phase of Sustainable Development Goals, climate change is certainly at the forefront of many key concerns. Understanding how climate change and future climate shocks will affect food security is crucially important and can be informed by this work examining how such shocks have already affected food security. We provide clear evidence across 15 countries that such shocks are associated with food insecurity and that productive capacities that assist smallholders in agricultural production may help to safeguard food security in the presence of these shocks. Furthermore, we show that fertilizers, pesticides, veterinary medicines, large livestock and assets may be particularly significant to alleviate food insecurity during or after a climate shock, though related agroecological practices could provide similar benefits. Efforts to foster food security resilience prior to a climate shock will also be important and our results also suggest this may be achieved through supporting the attainment of productive capacities and education.

Importantly, this provides several avenues in which climate change and food security must be considered in the future. First, development strategies must now build resilience and capacity for potential future shocks and impacts. Thus strategy needs to go beyond the traditional thinking focused on agricultural production- many of the SDGs are relevant to achieving poverty reduction and increasing education, which in turn can reduce food insecurities in the presence of a climate shock. Building adaptive capacity now may also provide mitigation benefits for the future [55]. Second, beyond shoring up smallholder farmers to withstand or cope with shocks in the future, strategies must consider responses following climate shocks. Recognizing that individual households will experience shocks in varying ways based on their adaptive capacities indicates that assessing “perceived shocks” is important. Household-level shocks may be more heterogeneous than large-scale disaster shocks that affect entire regions, providing new challenges for development work to identify and respond to such shocks that in some contexts may not be as obvious. However, we also believe that future work should more thoroughly analyze the kinds of shocks faced by households, their magnitude and potential impacts. Together, these perspectives provide a pathway under the new SDGs in which climate change and food security can be considered both directly and indirectly by achieving other development strategies.

## Supporting information

S1 File Supporting information. (DOCX)
