# Supplementary material for: A cross-country analysis of climate shocks and smallholder food insecurity
Source: PLoS One. 2018 Feb 23;13(2):e0192928. doi: 10.1371/journal.pone.0192928 (PMC5825057; doi:10.1371/journal.pone.0192928)
Supplement: S1 File — (DOCX) [file pone.0192928.s001.docx]

**Supplementary Information: Materials and Methods**

1. Sample structure

Data collection followed Climate Change, Agriculture and Food Security (CCAFS) protocols (<https://ccafs.cgiar.org/resources/baseline-surveys#household>). For visual presentation of descriptive means of focal variables, we aggregated sites and countries into the three regions displayed in Figure 1. However, statistical models adhered to the hierarchical structure of households clustered in sites and sites clustered in six regions (India, Nepal, Bangladesh, East Africa, West Africa, and Latin America). We disaggregated the countries of South Asia into separate regions because the three regions exhibited distinct differences across measures of food insecurity and the experience of climate shock (see Fig. 1) and also because models estimated notable differences in varying effects estimates for these regions; these effects allow for independent adjustments in baseline levels of the food insecurity outcome variable (see Fig. A).

2. Statistical model

CCAFS household surveys measured food insecurity as the total number of months in a typical year during which respondents reported not having adequate food for their household. Our research questions required the model to estimate the relative effects of predictor variables on the odds of more (or less) severe food insecurity. We therefore implemented binomial regression, which is fundamentally a model of the odds of a given outcome. The model estimates the following coefficients:

Varying (i.e., hierarchical, mixed, random effects) intercept effects at the household, site, and region level (see Main Text);

Fixed household-level effects of binary focal variables (shock, use of certified seed, use of fertilizer, use of pesticide, use of veterinary medicines, use of credit, upper 20% wealth index quantile, and lower 20% wealth index quantile);

Fixed household-level interaction effects of shock multiplied by each of the binary focal variables;

Fixed household-level effects on demographic control covariates (household size, whether or not the household was headed by a single male, whether or not the household was headed by a single female, three binary indicators of education level).

The model specification is reported in the main text.

We fitted the model using computational Bayesian methods because the hierarchical, nested nature of the data obligates the use of multilevel models [1], and Bayesian methods provide flexible and direct approaches to such multilevel applications, including the estimation of variance on varying effects parameters [2]. For model structure and prior specification see Gelman and Hill [3, p 381, 430].

Models were estimated using the R software environment [4-6] on the Sapper UNIX server at the UC Davis Social Science Data Service. The server is a Dell R820 32-core CPU with 512GB of RAM running Redhat Linux Enterprise version 6 (see <http://www.ssds.ucdavis.edu/computing>). The complete 600,000-iteration model run (300,000 burn-in, 300,000 posterior sample) lasted approximately 24 days. Model mixing and convergence were assessed by examining trace plots and kernel densities.

| **Table 1.** Coefficient estimates from the binomial model. The model produces raw household-level coefficient estimates (log-odds scale, col. 2) from the joint posterior, which are interpreted in the main text as odds ratios with 95% credibility intervals (cols. 3-5). The additive result of multiple coefficients (including interaction effects) describing the relative difference in odds of experiencing food insecurity are described in the main text and in Figure 3 in terms of hypothetical household scenarios. |
| --- |

| **Coefficient** | **Odds ratio** | **Estimate (log-odds)** | **Lower 95% CI** | **Upper 95% CI** |
| --- | --- | --- | --- | --- |
| Climate shock | 1.73 | 0.53 | 1.25 | 2.36 |
| Seed | 1.27 | 0.23 | 0.99 | 1.59 |
| Fertilizer | 0.85 | -0.17 | 0.63 | 1.12 |
| Pesticide | 0.65 | -0.43 | 0.51 | 0.83 |
| Veterinary meds | 0.96 | -0.05 | 0.74 | 1.21 |
| Credit | 1.24 | 0.20 | 0.88 | 1.71 |
| Wealthy | 0.82 | -0.21 | 0.62 | 1.07 |
| Poor | 1.71 | 0.53 | 1.33 | 2.16 |
| Lg. Livestock | 0.50 | -0.71 | 0.38 | 0.64 |
| Sm. Livestock | 1.06 | 0.05 | 0.82 | 1.36 |
| HH size | 1.02 | 0.02 | 1.00 | 1.05 |
| Single fem. HH | 0.98 | -0.03 | 0.82 | 1.14 |
| Single male HH | 1.02 | 0.00 | 0.73 | 1.36 |
| Education lev. 1 | 0.85 | -0.17 | 0.71 | 1.01 |
| Education lev. 2 | 0.78 | -0.25 | 0.64 | 0.94 |
| Education lev. 3 | 0.47 | -0.77 | 0.36 | 0.58 |
| Shock*seed | 0.81 | -0.22 | 0.61 | 1.06 |
| Shock*fertilizer | 0.85 | -0.18 | 0.61 | 1.14 |
| Shock*pesticide | 1.10 | 0.09 | 0.83 | 1.45 |
| Shock*vet.meds | 0.85 | -0.18 | 0.64 | 1.11 |
| Shock*credit | 0.80 | -0.25 | 0.53 | 1.18 |
| Shock*wealth | 0.77 | -0.27 | 0.55 | 1.03 |
| Shock*poor | 0.94 | -0.07 | 0.71 | 1.24 |
| Shock*lg.livstk | 1.71 | 0.52 | 1.27 | 2.23 |
| Shock*sm.livstk | 0.97 | -0.04 | 0.70 | 1.29 |

Figure A. Varying effects estimates predicting food insecurity from the binomial model. Estimates can be interpreted as independent adjustments in the baseline log-odds of experiencing more severe food insecurity specific to each level of clustering in the data; estimates are not translated to the odds scale due to their high degree of variance.

4. Supplementary references

1. McElreath R. Statistical Rethinking: A Bayesian Course with Examples in R and Stan Chapman & Hall/CRC Press; 2015.

2. Jackman S. Bayesian Analysis for the Social Sciences. United Kingdom: John Wiley & Sons, U.K; 2009.

3. Gelman A & Hill J. Data Analysis Using Regression and Multilevel/Hierarchical Models New York: Cambridge University Press; 2007.

4. Plummer M. JAGS: A program for analysis of Bayesian graphical models using Gibbs sampling. In Proceedings of the 3rd International Workshop on Distributed Statistical Computing (DSC 2003). Vienna, Austria. 2003.

5. Plummer M. rjags: Bayesian graphical models using MCMC. R package version 3.3.0. mcmc-jags.sourceforge.net. 2012.

6. R Core Team. R: A language and environment for statistical computing. R Foundation for Statistical Computing, Vienna, Austria. <http://www.R-project.org>. 2013.
